# Functional Characterization of Lamina X Neurons in *ex-Vivo* Spinal Cord Preparation

**DOI:** 10.3389/fncel.2017.00342

**Published:** 2017-11-01

**Authors:** Volodymyr Krotov, Anastasia Tokhtamysh, Olga Kopach, Andrew Dromaretsky, Yevhenii Sheremet, Pavel Belan, Nana Voitenko

**Affiliations:** ^1^Department of Sensory Signalling, Bogomoletz Institute of Physiology, Kyiv, Ukraine; ^2^Department of Molecular Biophysics, Bogomoletz Institute of Physiology, Kyiv, Ukraine; ^3^Chair of Biophysics and Molecular Physiology, Kyiv Academic University, Kyiv, Ukraine

**Keywords:** *ex-vivo* spinal cord preparation, lamina X neurons, electrophysiological recordings, primary afferent stimulation, retrograde labeling, Ca^2+^ imaging

## Abstract

Functional properties of lamina X neurons in the spinal cord remain unknown despite the established role of this area for somatosensory integration, visceral nociception, autonomic regulation and motoneuron output modulation. Investigations of neuronal functioning in the lamina X have been hampered by technical challenges. Here we introduce an *ex-vivo* spinal cord preparation with both dorsal and ventral roots still attached for functional studies of the lamina X neurons and their connectivity using an oblique LED illumination for resolved visualization of lamina X neurons in a thick tissue. With the elaborated approach, we demonstrate electrophysiological characteristics of lamina X neurons by their membrane properties, firing pattern discharge and fiber innervation (either afferent or efferent). The tissue preparation has been also probed using Ca^2+^ imaging with fluorescent Ca^2+^ dyes (membrane-impermeable or -permeable) to demonstrate the depolarization-induced changes in intracellular calcium concentration in lamina X neurons. Finally, we performed visualization of subpopulations of lamina X neurons stained by retrograde labeling with aminostilbamidine dye to identify sympathetic preganglionic and projection neurons in the lamina X. Thus, the elaborated approach provides a reliable tool for investigation of functional properties and connectivity in specific neuronal subpopulations, boosting research of lamina X of the spinal cord.

## Introduction

It has been established that neurons in the lamina X of the spinal cord play an important role in somatosensory integration (Ness and Gebhart, 1987), visceral nociception (Lantéri-Minet et al., 1993, 1995; Cervero and Laird, 2004; Eijkelkamp et al., 2007), autonomic regulation (Deuchars and Lall, 2015) and modulation of motoneuron outputs (Bertrand and Cazalets, 2011). Nevertheless, the predominant bulk of this knowledge relies on a set of morphological and immunohistochemical techniques while studies of functional properties of lamina X neurons remain rather exceptional. There have been electrophysiological studies of lamina X neurons (Bordey et al., 1996a,b; Bradaïa and Trouslard, 2002a,b; Bradaïa et al., 2004; Seddik et al., 2006, 2007), those focused largely on neuronal receptor function without investigating the neuronal connectivity and circuitry functioning. Moreover, studies of the lamina X neurons were predominantly performed using spinal cord slices (Phelan and Newton, 2000a; Bradaïa and Trouslard, 2002b) where ablating the dorsal and ventral roots disrupts genuine functional connectivity.

The LED oblique illumination technique, implemented for cell visualization in thick blocks of tissue (Safronov et al., 2007; Szücs et al., 2009), provides a unique opportunity to preserve the naïve tissue architecture along improved cell visualization, as demonstrated for various *ex-vivo* preparations, including the spinal cord of the desired tissue length (Safronov et al., 2007). The resolved cell visualization by this technique in *ex-vivo* spinal cord preparation was numerously confirmed in further studies of sensory input integration in the superficial dorsal horn (Szucs et al., 2013; Luz et al., 2015; Fernandes et al., 2016). Given all these benefits for the lamina X research, we used a combination of LED oblique illumination with *ex vivo* spinal cord preparation implementing also a longitudinal hemisection at the sagittal midline (Wilson et al., 2007, 2010; Hinckley et al., 2010; Meyer et al., 2010; Bui et al., 2013; García-Ramírez et al., 2014) for direct access to the area around the central canal.

In this article we describe the methodological approach of an *ex vivo* spinal cord preparation for functional studies of lamina X neurons and provide evidence of the reliable use of the preparation for electrophysiological recordings from individual lamina X neurons combined with primary fiber stimulations, calcium imaging and retrograde labeling of specific neuronal subpopulations to boost lamina X research.

## Materials and Methods

### Animals

Animals used in this study were P7–14 Wistar rats. Based on our experimental observations, use of elder animals (>P15) limited cell visualization in *ex vivo* spinal cord preparation.

This study was carried out in accordance with the European Commission Directive (86/609/EEC), the ethical standards of the International Association for the Study of Pain (IASP) and the Law of Ukraine on protection of experimental animals (N3447-IV, 21.02.2006). Experimental protocols were approved by the Animal Care and Use Committee at Bogomoletz Institute of Physiology (Kyiv, Ukraine) before the start of the research. All efforts were made to avoid or minimize suffering.

### *Ex-Vivo* Spinal Cord Preparation

**Figure 1:**
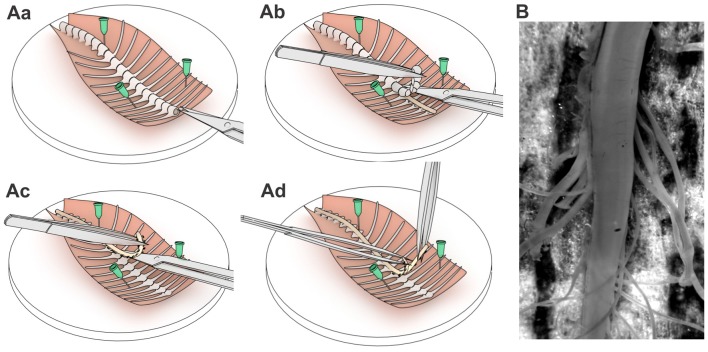
A step-by-step spinal cord tissue preparation procedure. Schematic illustrations of pinning the dissected spinal cord tissue **(Aa)**, retrieving the spinal cord **(Ab,Ac)** and exposing the lamina X area **(Ad)**. **(B)** The spinal cord tissue preparation with exposed lamina X area and both dorsal and ventral roots attached.

Animals were decapitated and vertebral column with attached ribs were cut out and placed in a Petri dish with Silgard bottom into sucrose solution that contained (in mM) 200 sucrose, 2 KCl, 1.2 NaH_2_PO_4_, 0.5 CaCl_2_, 7 MgCl_2_, 26 NaHCO_3_, 11 glucose (pH 7.4, 95% O_2_ and 5% CO_2_, room temperature). The dissected tissue was pinned on the dorsal side rostrally to the experimenter as depicted (Figure 1Aa) and the spinal cord was removed. For gentle removal we cut vertebrae with microscissors (Figure 1Ab) using an Olympus SZX7 stereo microscope and clipped the dorsal/ventral roots as close to the dorsal root ganglia as possible (Figure 1Ac). The dura matter was carefully cut above the ventral middle line and the spinal cord was gently split into two equal parts with two forceps (Figure 1Ad). The procedure typically took 5–6 min after the animal’s decapitation, enabling fast exposure of lamina X area to an oxygen-enriched medium. Afterwards, one part of the spinal cord was peeled from the remaining dura matter and superfluous roots (Figure 1B) and was glued to a gold plate to fix the tissue for experiments.

### Visualization of the Lamina X Neurons with LED Oblique Illumination

The number of studies performed in the longitudinally hemisected spinal cord utilized DIC for cell visualization, though using pups (P1–P4; Wilson et al., 2007, 2010; Hinckley et al., 2010; Meyer et al., 2010; Bui et al., 2013; García-Ramírez et al., 2014). We, however, failed in faithfully resolving cells in the area around the central canal with DIC when using the spinal cord preparations from P7–P14 rats. Given that a LED oblique illumination technique does not rely on transmitted light (Safronov et al., 2007; Szücs et al., 2009), we therefore, adapted LED oblique illumination to visualize neurons within the lamina X in our tissue preparation.

**Figure 2:**
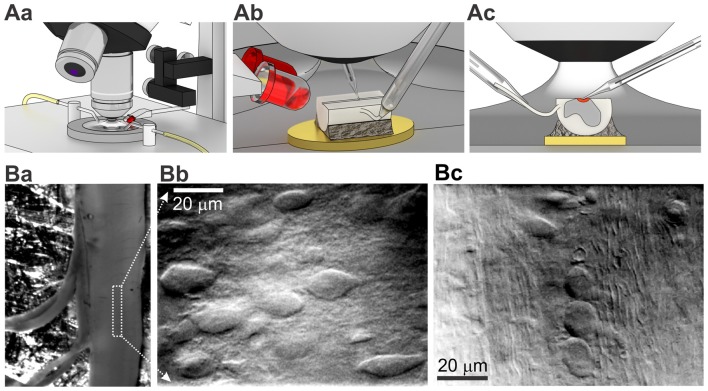
Visualization of lamina X neurons with infrared (IR) LED illumination in *ex-vivo* spinal cord tissue preparation. **(A)** Illustrations of optical system setup used for visualization and functional studies of intact lamina X neurons in spinal cord tissue preparation. The experimental setup includes an upright microscope, white and IR LEDs mounted on micromanipulator and 3D printed bath chamber **(Aa)**. Positioning of IR LED to adjust the best optical optimum for every *ex-vivo* spinal cord tissue preparation **(Ab)** before starting recordings from lamina X neurons (the area investigated is depicted as red, both electrodes are for recording and stimulation, yellow is a gold plate on which the tissue was fixed **(Ac)**. **(B)** Visualization of lamina X neurons in *ex-vivo* spinal cord preparation with attached L4 and L5 dorsal roots **(Ba)** within the area close to central canal (indicated by a white box) showing regions enriched with interneurons **(Bb)** and fibers traveling along sparse cells **(Bc)**, a sign of the boundary for the lamina X.

The *ex-vivo* spinal cord was placed in a 3D-printed plastic chamber filled with Krebs bicarbonate solution containing (in mM) 125 NaCl, 2.5 KCl, 26 NaHCO_3_, 1.25 NaH_2_PO_4_, 2 CaCl_2_, 1 MgCl_2_, 10 glucose and continuously bubbled with 95% O_2_ and 5% CO_2_ (pH 7.3). The perfusion rate was ~1.5–2 ml/min. As a source of light we used narrow beam infrared (IR) LED (860 nm, ±3°, SFH4550, Osram) attached to a manipulator (Figure 2Aa) that allowed to adjust an acute angle (10–20°) for each tissue preparation (Figure 2Ab) for the best cell visualization. The maximum of light intensity was acquired with OLY-150IR camera (Olympus, Japan) on upright microscope (BX50WI, Olympus, Japan) with a water immerse objective (60×, 0.90NA, Olympus, Japan). An additional white LED, placed parallel to the IR LED and coupled with 5× objective (Carl Zeiss, Jena, Germany), was used for positioning the tissue. With LED oblique illumination we enabled a resolved cell visualization within the lamina X area in *ex-vivo* spinal cord preparation from P7–14 animals (Figure 2B). The combined approach enabled visualizing cells below 50–70 μm from the tissue surface whereas gray matter surrounding the central canal could be easily distinguished in the middle of the preparation (Figure 2Ba). The lamina X neurons were clearly visualized in a higher magnification (Figure 2Bb) whereas surrounding areas contained numerous parallel fibers (Figure 2Bc).

### Electrophysiology of Lamina X Neurons in *ex-Vivo* Spinal Cord

#### Cell-Attached and Whole-Cell Recordings

Electrophysiological recordings were made from lamina X neurons in *ex vivo* spinal cords in physiological bicarbonate solution. All recordings were made at room temperature. Patch pipettes were pulled from borosilicate glass using a P-87 horizontal puller (Sutter Instruments, Novato, CA, USA) and had a resistance of 3–5 MΩ when filled with intracellular solution containing (in mM) 145 K-gluconate, 2.5 MgCl_2_, 10 HEPES, 2 Na_2_-ATP, 0.5 Na-GTP and 0.5 EGTA (pH 7.3). For detecting genuine neuron firing activity, cell-attached recordings from the lamina X neurons were conducted after formation of a gigaseal. Whole-cell recordings in current and voltage-clamp modes were made after rupturing the patch of membrane. Electrophysiological signals were acquired and filtered (Bessel, 2.6 kHz) using MultiClamp 700B amplifier (Molecular Devices, Sunnyvale, CA, USA) and digitized with Digidata 1320A under control of pClamp 9.2 software (Molecular Devices, Sunnyvale, CA, USA).

#### Dorsal and Ventral Root Stimulations

For electrophysiological recordings combined with root stimulation, a root was taken into a glass suction pipette with a tip size adjusted for each preparation (Figure 2Ac) and stimulated with square pulses of current generated by an ISO-Flex stimulator (A.M.P.I.). Dorsal roots were stimulated with 10–100 μA (50 μs duration) to activate low threshold A-fibers or 10–150 μA (1 ms duration) for activation of all primary afferents, including high threshold Aδ- and C-fibers. The stimulation frequency was 0.1 Hz to avoid rundown. Usually, we stimulated L5 dorsal (or ventral) root (otherwise L3–4) and recorded from cells in the same spinal segment.

#### Retrograde Labeling

Two distinct neuronal populations were distinguished within the lamina X area with retrograde labeling: preganglion sympathetic and projection neurons (Willis and Coggeshall, 2004; Deuchars and Lall, 2015). To visualize these specific lamina X neuronal subpopulations in *ex vivo* spinal cord, we used aminostilbamidine, an active compound of fluorogold dye.

##### Preganglionic sympathetic neurons labeling

For labeling preganglion sympathetic lamina X neurons, aminostilbamidine was injected intraperitoneally, similarly to previous studies (Ambalavanar and Morris, 1989; Anderson and Edwards, 1994). Briefly, aminostilbamidine (20 μl of 0.5% dissolved in saline) was injected using a syringe with 30G needle. The staining was observed in the lamina X after 2–3 days post-injection.

##### Projection neurons labeling

For labeling the projection lamina X neurons (Wang et al., 1999; Willis and Coggeshall, 2004), aminostilbamidine dye was injected into the lateral thalamus. Rat pups, anesthetized with a mixture of ketamine (30 mg/kg) and xylosine (2 mg/kg), were secured in stereotaxic apparatus. A skin on animal head was wiped with ethanol and one incision was made rostrocaudally along the midline to expose bregma and lambda anatomical points. An injection of aminostilbamidine (200 nl, 2%) was performed using a 1-μl syringe with 33G needle (WPI, USA) at the following coordinates: RC 2 mm, ML 2 mm, DV 3.8–4 mm. Then, skin was sutured and treated with Betadine (Egis, Hungary). Animals were kept on a warm pad (30°C) during the whole procedure and until full recovery from anesthesia. The aminostilbamidine staining was visualized in the lamina X 2–3 days after injection.

Imaging of labeled cells was performed with a FX1000 confocal microscope (Olympus, Japan) or epifluorescent upright BX50 microscope (Olympus, Japan) and a 12-bit cooled CCD camera (Sensicam PCO, Germany) combined with Polychrome IV monochromator (Till Photonics, Germany).

#### Propidium Iodide Staining

For the assessment of cell viability in the lamina X after tissue preparation and spinal cord hemisection, staining with probidium iodide (PI) was used. The tissue was incubated with 5 μM PI for 30 min, washed out (at least for 30 min) and then the image acquisition was performed.

#### Calcium Imaging

For intracellular calcium imaging in the lamina X neurons, we probed both membrane-permeable and -impermeable Fura 2 dye. The membrane-permeable Fura 2 acetoxylmethyl ester (AM) was bath loaded (5 μM in the presence of 0.2% pluronic F-127 for 1.5 h) and imaging was performed as described in details previously (Kopach et al., 2016). The membrane-impermeable Fura 2 pentapotassium salt (500 μM dissolved in EGTA-free internal solution) was loaded through a patch pipette (Kopach et al., 2011). Imaging was performed at least 10 min after membrane breakthrough to allow dye to diffuse intracellularly. The Ca^2+^ probe was excited at 340 and 380 nm using PolyChrome IV monochromator (Till Photonics, Germany); emission was measured at >510 nm using a 60×, NA 0.9 water-immersion objective (Olympus, Japan) and 12-bit cooled CCD camera (Sensicam, PCO, Germany) operated with TillVision software (Till Photonics, Germany). Analysis of changes in cytosolic free calcium concentration ([Ca^2+^]_i_) was performed as described before Kopach et al. (2016); [Ca^2+^]_i_ was expressed as the ratio of Fura 2 fluorescence at 340 and 380 nm (F_340_/F_380_) before and after stimulation.

#### Chemicals and Drugs

All chemicals and PI dye were obtained from Sigma-Aldrich (MO, USA). Aminostilbamidine, Fura 2 (pentapotasssium salt and AM ester) and pluronic F-127 were supplied by Thermo Fisher Scientific (MA, USA). Ketamine was manufactured by Farmak (Kyiv, Ukraine) and xylazine was purchased from Biovet Pulawi (Poland).

## Results

### Viability of Lamina X Neurons in *ex-Vivo* Spinal Cord Preparation

**Figure 3:**
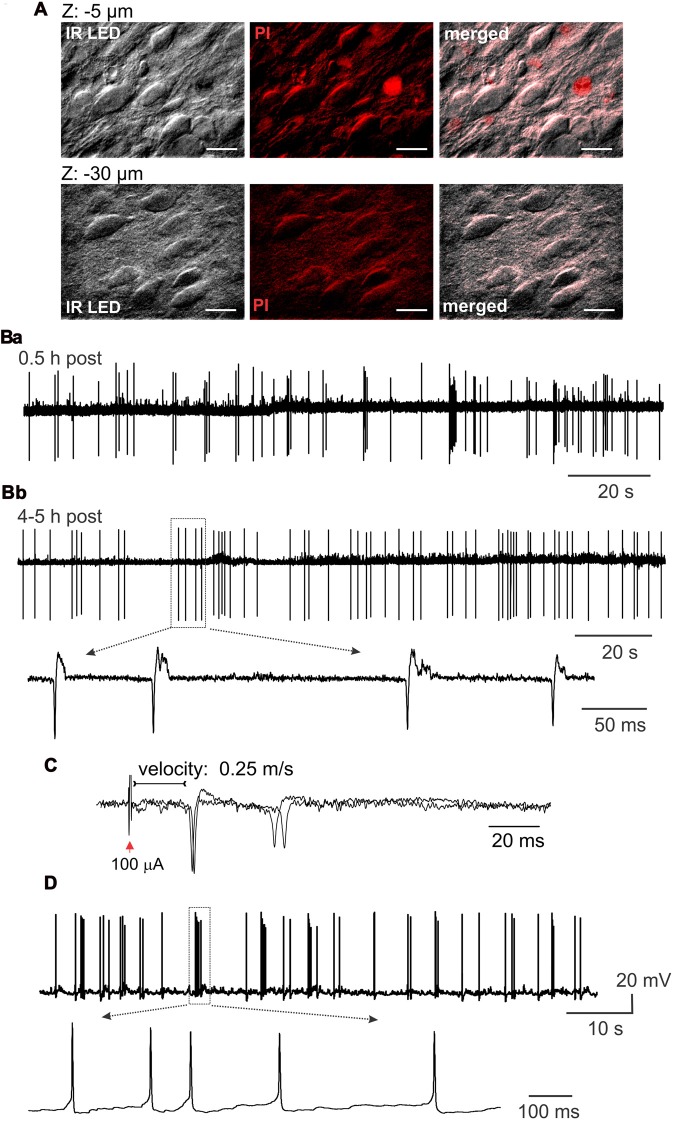
Lamina X neuronal viability in *ex-vivo* spinal cord tissue preparation. **(A)** Staining with propidium iodide, a marker of necrotic cell death, demonstrating a small number of damaged cells in the lamina X area in the spinal cord tissue preparation from P10 animal. Images are merged from IR LED oblique illumination and propidium iodide fluorescent signal taken at different focal planes from the surface of the preparation. Scale bars 20 μm. **(B)** Electrophysiological recordings made from the lamina X neurons in cell-attached mode, showing high neuronal activity in *ex-vivo* spinal cord shortly after the tissue preparation **(Ba)**, which remained at a stable-high level following 4–5 h after **(Bb)**. Insert showing individual spontaneous action potentials (sAP) generated by lamina X neurons. **(C)** Cell-attached recordings showing evoked AP generation after L5 dorsal root stimulation **(D)**. Whole-cell current clamp recordings from the lamina X neurons further proving high neuronal activity in *ex-vivo* spinal cord and functional viability of the cells for long lasting electrophysiological studies.

With the IR LED illumination, we visualized lamina X neurons in *ex-vivo* spinal cord by resolving a neuronal soma shape with contrasted membrane boundaries and determining fibers surrounding the lamina X (Figure 2B). To prove viability of the cells within the area, we used staining with propidium iodide (PI), a marker for necrotic cell death. We observed a small number of PI-positive cells in the lamina X located close to the surface of the tissue, whereas no PI-positive particles were detected in the deeper layers (>20 μm below the surface, Figure 3A).

### Electrophysiological Properties of Lamina X Neurons

The functional properties of lamina X neurons in *ex-vivo* spinal cord preparations were assessed by electrophysiological recordings made from individual cells. The recordings in cell-attached mode demonstrated high levels of activity of lamina X neurons with levels of frequency of firing remaining at a similar level shortly after the tissue preparation (0.5–1 h, Figure 3Ba) and over the course of experiment (around 4–5 h post-preparation, Figure 3Bb) in all neurons tested (*n* = 16). This demonstrates that *ex-vivo* spinal cords represent a viable tissue preparation for long lasting recordings in lamina X neurons. To prove that dorsal roots were not damaged by the preparation procedure, recordings of APs, evoked by primary afferent stimulation, were performed. The evoked APs were recorded in lamina X neurons (12 out of 21 cells tested), demonstrating the reliable use of the preparation for studies of synaptic transmission between primary afferents and lamina X neurons. The analysis of the latencies for evoked APs revealed that the estimated conductance velocity was less than 0.5 m/s, the value corresponding to the C-fiber conductance rate (Figure 3C). The recordings in current clamp configuration also confirmed high spontaneous action potential (sAP) activity of lamina X neurons (Figure 3D). Together, these results demonstrate the functional viability of *ex-vivo* spinal cord preparation for long lasting investigation of lamina X neurons for testing the patterns of their spontaneous and evoked firing activity.

**Figure 4:**
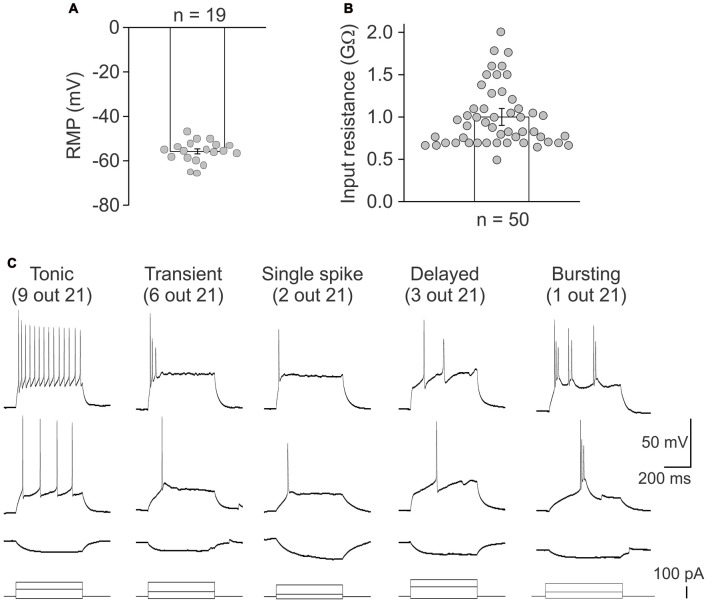
Heterogeneity of lamina X neurons by firing pattern discharges. Electrophysiological properties of lamina X interneurons for the resting membrane potential **(A)** and the input resistance **(B)**. **(C)** Different patterns of discharge revealed by lamina X neurons in response to current injections of increasing amplitude.

Whole cell recordings further proved the functional viability of lamina X neurons in *ex-vivo* spinal cord preparation. The resting membrane potential of the tested neurons was −56 ± 1 mV (*n* = 19; Figure 4A) and the average input resistance was 1.0 ± 0.1 GΩ (*n* = 50, Figure 4B). Such high resistance value requires the consideration of possible leak currents since it may produce large cell depolarizations. This is particularly critical for recordings in current clamp mode since a shift in the resting membrane potential in 5–7 mV-range may cause switching neuronal firing to a sustained, non-physiological pattern.

Firing patterns are a widely used characteristic for classifying the superficial dorsal horn interneurons (Punnakkal et al., 2014; Kopach et al., 2015, 2017). Therefore, it may be useful for characterizing lamina X neurons. We addressed this by recording a neuronal discharge elicited by exogenous current injections. We observed several different patterns of neuronal discharge in the lamina X neurons (Figure 4C) those included the tonic firing (recorded in 9 lamina X neurons out of 21 tested), transient (6/21), single spiking (2/21), delayed (3/21) and bursting firing (1/21). Such heterogeneity of firing patterns of lamina X neurons assumes heterogeneous neuronal properties amongst this population and may suggest their distinct functional roles within the circuitry.

**Figure 5:**
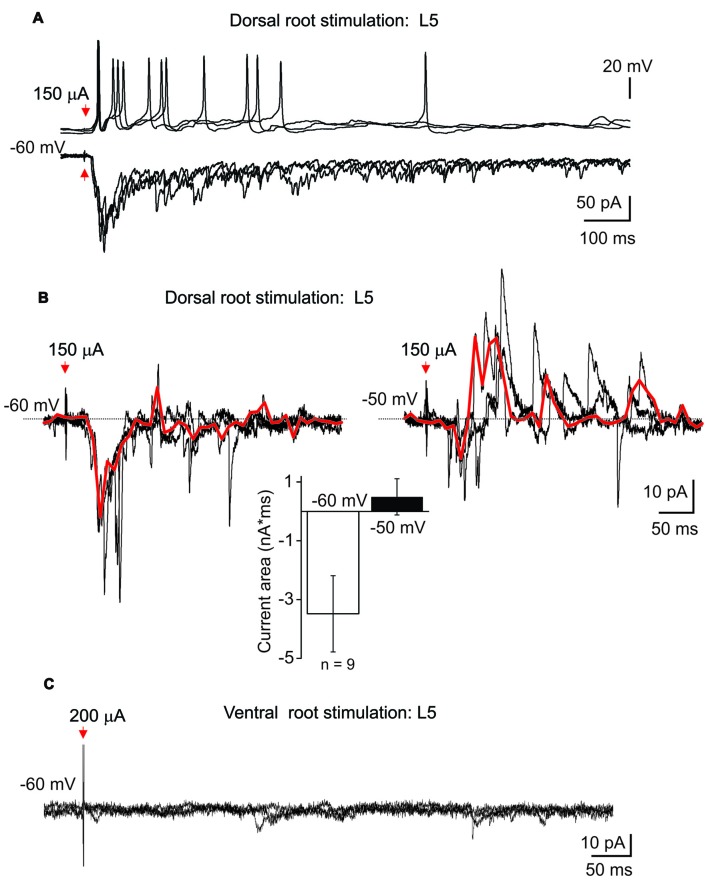
Postsynaptic currents evoked in lamina X interneurons by root stimulation. **(A)** Recordings of postsynaptic currents induced in lamina X interneuron in response to L5 dorsal root stimulation at −60 mV. Top row: changes in the membrane potential in lamina X neurons during primary afferent stimulation (50 μs/100 μA). **(B)** Examples of inhibitory polysynaptic currents recorded from the same lamina X neuron at different membrane potentials in response to primary afferent stimulation (1 ms/150 μA) of L5 dorsal root and summary of mean current area at different potentials (*n* = 9 cells;* p* < 0.05 paired *t*-test). Red line represents the average of the trials illustrated. **(C)** The absence of any responses of lamina X neurons in response to L5 ventral root stimulation.

To figure out the innervation of lamina X neurons by dorsal and ventral roots, whole-cell recordings in *ex-vivo* spinal cord were combined with root stimulations. The stimulation of L5 dorsal root elicited postsynaptic currents in all tested lamina X neurons (*n* = 20); direct primary afferent inputs were distinguished by low jittering and high reproducibility of the responses (Figure 5A). Strong excitatory responses induced the generation of APs that could be detected in current clamp mode (Figure 5A upper row). The dorsal root stimulation elicited polysynaptic inhibitory currents in nine tested cells (Figure 5B). Given that a reversal potential for chloride anions was approximately −80 mV, producing a low anion flow within the resting membrane potential range, changing the membrane potential to −50 mV was required for reliable detection of inhibitory currents (Figure 5B). Stimulation of the L5 ventral root, however, failed to evoke any response (APs or postsynaptic currents) in all tested neurons (0 out of 20 tested; Figure 5C). This may indicate a lack of reciprocal synaptic connections between lamina X neurons and the neurons sending axons through the L5 ventral root.

### The Depolarization-Induced Changes in [Ca^2+^]_i_ in Lamina X Neurons

**Figure 6:**
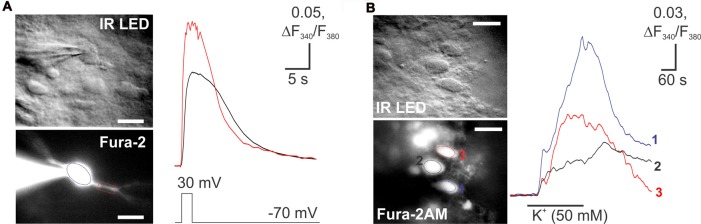
Imaging of changes in cytosolic free calcium concentration in lamina X neurons. **(A)** Images of lamina X neuron loaded with a membrane-impermeable Fura 2 Ca^2+^ dye, through the patch pipette (blue circle) and rise in [Ca^2+^]_i_ in lamina X neuron in response to membrane depolarization (voltage step from −70 mV to 30 mV for 2 s duration). **(B)** Images of lamina X neurons loaded with Fura 2/AM, a membrane-permeable Ca^2+^ dye (left) [Ca^2+^]_i_ in response to membrane depolarization with high-potassium medium (KCl 50 mM). Changes in [Ca^2+^]_i_ represented as changes in the ratio of Fura 2 fluorescence at 340 nm to 380 nm. Scale bars 20 μm.

In spite of the fact that fluorescent imaging techniques represent a powerful tool for investigating the neuronal function, calcium imaging has not been used in the lamina X neurons. Therefore, pilot studies were necessary to prove a capability of our tissue preparation for the calcium imaging techniques in lamina X neurons. We probed both membrane-permeable and -impermeable forms of a calcium dye Fura 2 for detecting the changes in [Ca^2+^]_i_ during depolarization of lamina X neurons. A robust [Ca^2+^]_i_ rise was detected in lamina X neurons when depolarizing cells with a voltage step (from −70 mV to 30 mV; Figure 6A) or during application of high potassium medium (50 mM; Figure 6B). The [Ca^2+^]_i_ rise was transient and recovered back to basal level, indicating a physiological intracellular calcium regulation that further confirms the viability of the lamina X neurons in *ex vivo* tissue preparation.

### Electrophysiological Properties of Lamina X Neuron Subpopulations

It is known that the area around the central canal of the spinal cord contains at least two functionally distinct neuronal populations, sympathetic preganglion (SPG; Deuchars and Lall, 2015) and projection (PN) neurons (Willis and Coggeshall, 2004). Given that, we performed identification of these neuronal subtypes in *ex-vivo* spinal cord preparation with aminostilbamidine, an active compound of Fluorogold, to test them electrophysiologically.

**Figure 7:**
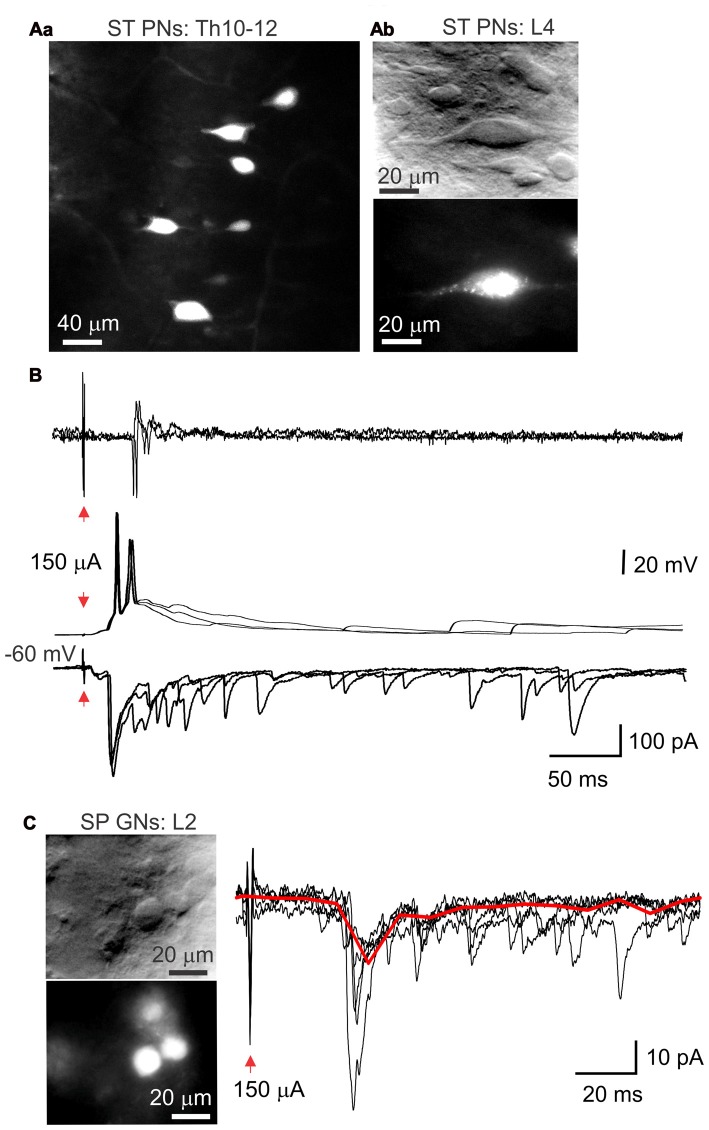
Subpopulations of lamina X neurons identified with retrograde labeling. **(A)** Images of spino-thalamic projection interneurons (ST PNs) in the lamina X in the upper thoracic **(Aa)** and lumbar **(Ab)** spinal segments labeled retrogradelly with aminostilbamidine dye injected into lateral thalamus. Image on **(Aa)** represents cumulated Z-stack of 20 μm with 2-μm step. **(B)** Electrophysiological recordings made from ST PN in the lamina X in cell-attached (upper row) and whole-cell configuration (middle and bottom), showing functional responses with stimulation of L4 dorsal root. **(C)** IR LED and fluorescent images of sympathetic preganglion neurons (SP GNs) in lamina X in L2 spinal cord segment and whole-cell recordings made from a SP GN, showing postsynaptic currents evoked by stimulation of L3–4 dorsal root (1 ms/150 μA). Red line represents the average of the trials illustrated.

Injection of aminostilbamidine into the lateral thalamus caused fluorescent labeling of spino-thalamic lamina X PNs (Figures 7Aa,Ab), which were detected through all spinal segments tested (Th5-L6) on the side contralateral to injection. Electrophysiological recordings made from the labeled cells in current and voltage clamp modes revealed strong innervation by primary afferent fibers (Figure 7B) and the characteristic bursting firing pattern (Figure 4C).

Aminostilbamidine injected intraperitoneally produced a robust staining of SPG neurons in lamina X in *ex-vivo* spinal cord (Figure 7C). We observed labeled neurons in thoracic and higher lumbar spinal segments (L1-L2), as expected. Patch clamp recordings revealed that SPGNs displayed the delayed firing pattern (Figure 4C). Primary afferent stimulation elicited excitatory postsynaptic currents in SPGNs (Figure 7C), however the current amplitude was not high enough to induce AP generation.

## Discussion

Spinal cord lamina X neurons are key integrators for visceral somatosensory and nociceptive inputs and play an important role in autonomic regulation and motoneuron output modulation. However, the functional properties of lamina X neurons and their connectivity remain poorly understood because of limited access to the area around the central canal. In this work we introduce a methodological approach that combines an *ex-vivo* spinal cord preparation and LED oblique illumination to implement electrophysiology and imaging techniques in the lamina X. Using both imaging and electrophysiology we demonstrate tissue viability for long lasting studies in the lamina X area and demonstrate electrophysiological characteristics of some of the lamina X neurons.

Over the recent decades there have been electrophysiological studies of lamina X neurons those, however, were exceptional and carried out in the spinal cord slices to enable cell visualization with transmitted light (Bordey et al., 1996a,b; Phelan and Newton, 2000a,b; Bradaïa and Trouslard, 2002a,b; Bradaïa et al., 2004; Bertrand and Cazalets, 2011). Meanwhile, a number of benefits has been discovered when using thick blocks of spinal cord tissue rather than a few-hundred micron slices. To overcome the optical limitations in thick tissue, LED oblique illumination had been successfully adopted (Safronov et al., 2007; Szücs et al., 2009). In addition to the improved cell visualization, this approach provides a set of physiological benefits, including preserved longitudinal architecture (e.g., intersegmental connections, from cervical to sacral segments) and attached dorsal and/or ventral roots that are essential for investigating the neuronal connectivity and vertical circuitry architecture of the spinal cord. Bearing all this in mind, we implemented the IR LED illumination to visualize cells in the area around the central canal following the spinal cord hemisection carried along the medial line. A hemisection of the spinal cord is routinely used throughout the studies and does not compromise tissue viability in different spinal cord laminae (Wilson et al., 2007, 2010; Hinckley et al., 2010; Meyer et al., 2010; Bui et al., 2013; García-Ramírez et al., 2014). Ultimately, such methodological combination provided us with a possibility of contrasted visualization of lamina X neurons with LED oblique illumination for investigating the functional properties of these neurons in *ex vivo* spinal cord. On the other hand, major limitation of LED illumination is the age of experimental animals. Similarly to the previous reports (Safronov et al., 2007; Szücs et al., 2009), cell visualization was obstructed in the lamina X area when using animals aged > P15 due to increased density of myelinated fibers. This may be an issue for some of the specified studies given the ongoing synaptogenesis (Weber and Stelzner, 1980; Fitzgerald et al., 1994; Li and Zhuo, 1998; Bardoni, 2001) and receptor subunit compositions varying with aging (Jakowec et al., 1995a,b; Brown et al., 2002).

A set of technical approaches implemented for visually-guided electrophysiology in the lamina X area allowed us to assess the functional properties of lamina X neurons in *ex-vivo* spinal cord. Functional viability of lamina X neurons in the spinal cord preparation was proved by their firing activity, which remained stable over few hours, and by consistent neuronal responses to primary afferent stimulation (L5 dorsal root). Strikingly, we failed to detect innervation of lamina X neurons in L5 spinal segment when stimulating L5 ventral root. This is very likely due to the absent reciprocal connections between lamina X neurons and the neurons projecting through L5 ventral root. Similarly to the superficial dorsal horn area (Yasaka et al., 2010; Punnakkal et al., 2014; Kopach et al., 2015, 2017), different patterns of discharge were observed for the lamina X neurons, indicating their heterogeneity. Indeed, at least three functionally distinct neuronal types were identified in the lamina X, those were determined as central pattern generator neurons (Bertrand and Cazalets, 2011), preganglion sympathetic (Deuchars and Lall, 2015) and projection neurons (Willis and Coggeshall, 2004). Our electrophysiological recordings confirmed the distinct patterns of firing discharge for two of those neuronal subpopulations, distinguished with a retrograde labeling. A characteristic bursting firing pattern of PNs was consistent to that for PNs in lamina I (Ruscheweyh et al., 2004). Together, this argues towards defining the subpopulations of lamina X neurons by their electrophysiological properties (e.g., firing activity).

To the best of our knowledge, calcium imaging techniques have not been applied yet in the lamina X area, which could cast light on intracellular calcium signaling pathways for regulating lamina X neuronal function. Given that, we have also probed the *ex vivo* spinal cord preparation using intracellular calcium imaging in lamina X neurons by testing out ratiometric fluorescent calcium dye Fura 2 in both membrane-permeable (bath loaded) or -impermeable forms (loaded through a patch pipette). The robust [Ca^2+^]_i_ rise in response to depolarization of lamina X neurons was similar to those in other types of neurons (Kopach et al., 2011), confirming its reliable application in the lamina X neurons in our tissue preparation. The use of tagged dextran (Blivis and O’Donovan, 2012) is another fluorescent technique that might be implemented in *ex-vivo* spinal cord for exploring neuronal connectivity within the lamina X or for mapping parasympathetic preganglionic neurons (by loading dextran through ventral roots). Genetically modified animals further serve as promising tool to expand the lamina X research, e.g., rats expressing GFP-conjugated c-fos (Cruz et al., 2013), a marker of neuronal activity, upregulated in lumbar lamina X in visceral pain models (Lantéri-Minet et al., 1995; Eijkelkamp et al., 2007) would be useful in visceral nociception studies. Alternatively, the tissue preparation may spare desired peripheral nerves (Li and Baccei, 2017). Since some of them, for instance, intercostal or pelvic splanchnic nerves, innervate exclusively viscera, nerve stimulation would allow to distinguish the lamina X neurons involved in processing of somatosensory and visceral nociceptive inputs.

Summarizing, the proposed methodological approach for investigating the lamina X neurons represents a reliable tool for functional studies in the lamina X, including both imaging and electrophysiological techniques, for long lasting assessment of neuronal activity and functional properties in *ex-vivo* spinal cord. The reliable and feasible tissue preparation will potentially advance research of the lamina X area of the spinal cord.

## Author Contributions

VK: concept of the study, research design, electrophysiological recordings, fluorescent calcium imaging, confocal imaging, animal surgeries and injections, data analysis and interpretation, manuscript preparation. AT: spinal cord preparations, electrophysiological recordings, data analysis. AD: design and installation of LED oblique illumination, technical supervision, preparation of figures. YS: confocal imaging. OK: data interpretation, preparation of figures, manuscript preparation and critical revision. PB and NV: conceiving the study, manuscript revision.

## Conflict of Interest Statement

The authors declare that the research was conducted in the absence of any commercial or financial relationships that could be construed as a potential conflict of interest.
